# Impact of emotion regulation on psychosocial and emotional problems in Dutch adolescents with inflammatory bowel disease

**DOI:** 10.1002/jpr3.70299

**Published:** 2026-11-03

**Authors:** Sandra Jansen‐Kamphorst, Romy Snetselaar, Juliette Greidanus, Esther Eva Hartman, Miriam van Pelt, Janneke Stapelbroek, Brigitte Vugs

**Affiliations:** ^1^ Department of Medical Psychology Catharina Hospital Eindhoven The Netherlands; ^2^ Uniek kind‐jeugdpraktijken Utrecht The Netherlands; ^3^ Department of Medical Psychology Jeroen Bosch Medical Center Den Bosch The Netherlands; ^4^ Department of Medical and Clinical Psychology Center of Research on Psychological and Somatic Disorders (CoRPS), Tilburg University Tilburg The Netherlands; ^5^ Department of Paediatrics Catharina Hospital Eindhoven The Netherlands; ^6^ Department of Medical Psychology Máxima Medical Center Eindhoven The Netherlands

**Keywords:** maladaptive coping, mental health screening, pediatric gastroenterology, Strengths and Difficulties Questionnaire (SDQ)

## Abstract

**Objectives:**

Adolescents with inflammatory bowel disease (IBD) are at increased risk of psychosocial and emotional problems. This study examined the role of emotion regulation (ER) to (1) determine the prevalence of psychosocial and emotional problems in adolescents with IBD, (2) examine associations with sociodemographic and clinical characteristics, and (3) evaluate associations with ER strategies.

**Methods:**

Adolescents (ages 12–17 years) with IBD from three Dutch medical centers completed routine psychosocial screening. Psychosocial and emotional problems were assessed with the Strengths and Difficulties Questionnaire (SDQ). Norm comparisons were performed using one‐sample *t*‐test on raw SDQ scores, and prevalence estimates were based on age‐ and sex‐specific percentile scores. ER strategies (adaptive, maladaptive) were measured with the Dutch FEEL‐KJ. Sociodemographic and clinical data were obtained from medical records/questionnaires. Associations were examined using Spearman correlations and stepwise multiple regression, with psychosocial and emotional problems as outcomes.

**Results:**

Compared with Dutch population‐based norms, adolescents with IBD reported significantly higher psychosocial and emotional problems than reference norm groups (*p* < 0.001). Elevated scores were observed in 64.3% for total difficulties and 42.9% for emotional problems. Maladaptive ER showed strong positive associations with both outcomes. Sociodemographic and clinical characteristics were not significantly associated with psychosocial or emotional problems.

**Conclusions:**

Maladaptive ER is a prominent correlate of psychosocial and emotional problems in adolescents with IBD, whereas background characteristics were not supported. Targeting dysfunctional ER strategies may facilitate early identification of at‐risk adolescents and guide timely interventions.

## Abstract (highlights)

**What is Known:**

- Internalizing psychological problems (depression and anxiety) are more common in adolescents with inflammatory bowel disease (UBD) than in the general population.
- Several demographic and clinical factors have been identified as related to the increased risk of psychosocial problems; however, these have never been studied in conjunction before.

**What is New:**

- This is the first study to examine multiple potential risk factors in multifactorial approach, including emotion regulation (ER) strategies.
- These findings show that maladaptive ER strategies are strongly associated with higher psychosocial and emotional problems in adolescents with IBD, which can help identify those at greatest risk.
- These results could support the development of targeted interventions aimed at improving ER strategies in adolescents with IBD.

## 1 INTRODUCTION

The burden of living with inflammatory bowel disease (IBD), which includes the conditions ulcerative colitis (UC), Crohn's disease (CD), and IBD unclassified (IBD‐U), affects adolescent psychological development.^1^ Children with chronic diseases generally experience more daily stressors than healthy children.^2^ The chronic and unpredictable nature of IBD can be challenging during adolescence, a developmental period marked by increasing autonomy and changing social demands.^3^, ^4^ A meta‐analysis showed that psychosocial problems, especially emotional problems are more prevalent among adolescent with IBD.^5^

Emotional problems refer to internal experiences such as anxiety, low mood, excessive worrying, and psychosomatic complaints, whereas psychosocial problems encompass a broader range of difficulties in emotional, behavioral, and social functioning.^6^ Previous studies have predominantly focused on emotional difficulties, such as anxiety and depression.^7^ However, IBD may also affect broader aspects of psychosocial functioning, including school participation, peer relationships, and everyday social functioning, although these domains have received less systematic attention.^8^, ^9^, ^10^ Examining both emotional and broader psychosocial problems may therefore provide a more comprehensive understanding of adolescent functioning in IBD.

Several studies on the sociodemographic factors and clinical characteristics in IBD have identified potential risk factors for psychosocial problems in patients.^11^, ^12^, ^13^, ^14^ Girls and older adolescents may be more at risk,^11^, ^14^ and high disease activity or recent diagnosis can further increase risk.^12^, ^13^ As with other chronic diseases, children with IBD growing up in families with low socioeconomic status (SES) may also be at increased risk of psychosocial problems.^14^ These sociodemographic and clinical factors provide important context for understanding which adolescents may be at increased risk of psychosocial problems. However, findings remain inconsistent, and potentially modifiable psychological factors have received less attention.

Emotion regulation (ER) is one such modifiable psychological factor that may help explain how adolescents manage ongoing disease‐related stressors during this vulnerable developmental period. ER refers to the ability to monitor and modulate emotional experiences and emotional expression.^15^ The use of dysfunctional ER strategies has been consistently linked to the development of psychosocial problems.^2^, ^15^, ^16^ ER strategies can broadly be categorized as either adaptive or maladaptive.^17^ Adaptive strategies, such as acceptance, problem solving, and seeking social support, generally support goal‐directed functioning and socio‐emotional well‐being. In contrast, maladaptive strategies, such as rumination, catastrophizing, and avoidance, are more likely to hinder effective coping and are more often associated with psychological problems.^18^ During adolescence, when emotional demands increase and regulation becomes more autonomous,^4^ ER may play an important role in emotional and psychosocial functioning.^18^

Previous studies of children with other chronic diseases have shown that they experience more stressors ^1^ and therefore are at greater risk of using maladaptive ER strategies.^19^, ^20^, ^21^ However, to our knowledge, ER strategies have not yet been investigated in children with IBD. In addition, risk factors for psychosocial and emotional problems are likely interrelated and may even reinforce one another, making the likelihood of serious problems greater than expected from the sum of individual risk factors.^22^ This exponential accumulation of risk is referred as the cumulative risk model (CRM),^23^ and it may also be relevant in IBD, where multiple sociodemographic, clinical, and psychological risk factors can co‐occur. The current study aimed to determine the prevalence and severity of psychosocial and emotional problems in adolescents with IBD 12–17 years, assess their associations with sociodemographic and clinical characteristics and ER strategies, and to examine the effect of cumulative risk.

A better understanding of these emotional and psychosocial problems and their associated risk factors may support earlier identification and guide timely interventions.

Our specific research questions were:

(1) What is the prevalence and severity of psychosocial and emotional problems among adolescents (aged 12–17 years) with IBD?
(2) Which sociodemographic characteristics (sex, age, and SES), clinical characteristics (disease duration, disease activity), and ER strategies (adaptive and maladaptive) are associated with psychosocial and emotional problems?
(3) What is the effect of the exponential accumulation of risk using the CRM?

## 2 METHODS

### 2.1 Ethics statement

The study was approved by the Medical Ethical Committee of the Maxima Medical Center (N23.043) as not subject to the Dutch Medical Research Involving Human Subjects Act (WMO). Secondary approval was obtained from the participating centers. The Tilburg University Ethics Review Board conducted a data management review (TSB RP1848). All participants and their parents or legal guardians were informed about the study and provided informed consent.

### 2.2 Study design and study population

The Dutch Medical Federation established a best‐practice guideline for pediatric care in all children and adolescents with IBD,^24^ prescribing biannual screenings for psychosocial and emotional problems. In this cross‐sectional study, we used data collected from these standard screenings. Data were collected from the Catharina Hospital in Eindhoven (since February 2021), the Maxima Medical Center in Veldhoven, and the Jeroen Bosch Hospital in Den Bosch (since February 2023) until December 2024. A total of 105 adolescents were invited to complete the questionnaires, of whom 76 returned them. Six patients were excluded due to incomplete responses, resulting in a final sample of 70 adolescents (Table 1). Psychiatric comorbidity was obtained from the patients' medical records and was defined as previously established DSM‐classified diagnoses (mainly including attention deficit/hyperactivity disorder, autism spectrum disorder, depressive disorders, and anxiety disorders). In addition to the standard questionnaires, patients were invited to complete supplementary questionnaires evaluating adaptive and maladaptive ER strategies. Most participants completed these questionnaires, though numbers varied slightly between subscales (Table 1). Adolescents completed the questionnaires on paper independently, as part of the standard care procedures. They were administered additional questionnaires to evaluate ER strategies. Adolescents were eligible for inclusion in this study if they were diagnosed with IBD according to the revised Porto criteria,^27^ were between 12 and 17 years of age, and had sufficient knowledge of the Dutch language to complete questionnaires. The final sample represents 67% of the adolescents invited.

**Table 1:** Participant characteristics.

	*N*	Median (IQR)	Min	Max
Age (years)	70	15.6 (1.8)	12.1	17.8
Sex				
Female	46 (65.7%)			
Male	24 (34.3%)			
Psychiatric comorbidity				
Present	25 (35.7%)			
Absent	45 (64.3%)			
Socioeconomic status	70	0.15 (0.23)	−0.423	1.000
Disease duration (years)	68	0.95 (3.0)	0.0	11.8
Disease activity^a^	66			
Remission	36 (54.5%)			
Mild	24 (36.4%)			
Moderate–severe	6 (9.1%)			

*Note*: Data on disease activity were available for 66 patients. Abbreviation: IQR, interquartile range.

^a^ The disease activity for Crohn's disease was measured with the Pediatric Crohn's Disease Activity Index, where an index score of <10 = in remission, 10–27.5 = mild disease activity, and ≥30 = moderate/severe disease activity.^25^ The disease activity for ulcerative colitis and IBD‐U was measured with the Pediatric Ulcerative Colitis Activity Index, where an index score of <10 = in remission, 10–34 = mild disease activity, and ≥35 = moderate/severe disease activity.^26^ Moderate and severe disease activity categories were combined due to small group sizes.

### 2.3 Definitions

#### 2.3.1 Psychosocial and emotional problems

Psychosocial and emotional problems were measured using the 25‐item self‐report Strengths and Difficulties Questionnaire (SDQ).^28^, ^29^ Details on the subscales, scoring, and Likert scale are provided in Supporting Information S1: Table S1. For this study, psychosocial problems were measured using the Total Difficulties score, and emotional problems using the Emotional Symptoms subscale. Dutch normative data from a self‐report general population sample of adolescents aged 12–17 years were available from Vugteveen et al.^29^ Age‐ and sex‐specific percentile scores were derived using Dutch normative data as reported by Vugteveen et al.^29^ and the Dutch SDQ scoring manual.^30^ Percentile scores were used to classify scores as normal or elevated and to describe prevalence. Raw scores were used for statistical analyses.

#### 2.3.2 Participant characteristics

Sociodemographic characteristics were assessed with a study‐specific questionnaire, which included sex (male or female), age, and SES.

SES was assessed using scores defined by Statistics Netherlands.^31^ These scores are based on household data concerning welfare (a combination of income and wealth), the highest level of education, and recent labor participation, with an average score of 0. A positive score reflects higher‐than‐average status, while a negative score reflects lower‐than‐average status (range: –2 to 1).

The clinical characteristics included disease duration (in years) and disease activity, which were obtained from patients’ medical records. For patients with CD, the short Pediatric Crohn's Disease Activity Index (PCDAI) was used, with scores classified as remission (<10), mild (10–27.5), moderate (30–37.5), or severe (>37.5).^25^ In patients with UC and IBD‐U, the Pediatric Ulcerative Colitis Activity Index (PUCAI) was used, with scores classified as remission (<10), mild,^10^, ^11^, ^12^, ^13^, ^14^, ^15^, ^16^, ^17^, ^18^, ^19^, ^20^, ^21^, ^22^, ^23^, ^24^, ^25^, ^26^, ^27^, ^28^, ^29^, ^30^, ^31^, ^33^, ^34^, ^35^ moderate^32^ ^–64^, or severe (>65).^26^ Moderate and severe disease activity categories were combined due to small group sizes. Both the PCDAI and PUCAI are considered reliable instruments.^33^, ^34^ A score of 10 or higher was subsequently coded as a risk factor in the cumulative risk score.

### 2.4 ER strategies

The Dutch translation of the self‐report *Fragebogen zur Erhebung der Emotionsregulation bei Kindern und Jugendlichen* (FEEL‐KJ) was used to measure ER strategies.^32^, ^35^ The FEEL‐KJ consists of 90 statements about how an adolescent copes with anger, sadness, and fear on a 5‐point Likert scale. In this study, we used two summary scales: adaptive and maladaptive.^35^ Cut‐off scores were based on the normative guidelines of the FEEL‐KJ manual. A T‐score >60 was used to indicate maladaptive ER strategies, whereas a T‐score <40 was used to indicate adaptive ER strategies.^35^

### 2.5 Cumulative risk exposure

An exponential model may account for the increased associations between risk accumulation and youth well‐being.^36^ Snetselaar et al.^23^ introduced the distinction between “additive” and “cumulative” risk, albeit in a different context. Additive risk refers to the sum of dichotomized risk factors (previously and typically referred to as cumulative risk), whereas cumulative risk refers to the squared score of that sum to account for an exponential growth effect.

For the current study, all independent variables were binary coded as 0 (no risk) or 1 (risk). Risk factors were defined as follows: sex (boy = 0, girl = 1), age (≤14 = 0, ≥15 = 1), SES (≥0 = 0, <0 = 1), disease duration (≥6 months = 0, <6 months = 1), disease activity (PUCAI/PCDAI score <10 = 0, ≥10 = 1), maladaptive ER strategies (≤60 = 0, >60 = 1), and adaptive ER strategies (≥40 = 0, <40 = 1). Cut‐off values were based on established clinical thresholds and prior literature as described in the Introduction.

### 2.6 Statistical analysis

Analyses were conducted in IBM SPSS Statistics (v29).^37^ *p*‐values < 0.05 were considered statistically significant.

Missing data were inspected at the item and scale level. When <20% of items were missing within a scale/domain, person‐mean imputation was applied within that scale; otherwise, cases were treated as missing for that scale to ensure the validity and reliability of the results.^38^

Differences from Dutch normative mean scores reported by Vugteveen et al. were examined using one‐sample *t*‐test on raw SDQ scores. Prevalence was described as the proportion of adolescents with elevated psychosocial and emotional problems, defined as scores at or above the 80th percentile based on age‐ and sex‐specific Dutch normative data.^29^

Due to non‐normal distributions, Spearman's rank correlations were used. Variables showing significant independent associations in the correlation analyses were entered as predictors into stepwise multiple regression analyses to evaluate their independent associations with psychosocial and emotional problems. Correlations and regressions were conducted on raw SDQ scores.

Assumptions for the regression models were evaluated, including linearity, influential outliers, multicollinearity (variance inflation factors), and the normality and homoscedasticity of residuals.

## 3 RESULTS

### 3.1 Study population

A total of 70 patients with IBD were eligible (CD = 33, UC = 30, IBD‐U = 7). The sample included more girls (65.7%, *n* = 46) than boys. The median disease duration was 0.95 years (range: 0.0–11.8). Disease activity data were available for 66 patients. The majority of patients were in remission or had mild disease activity (*n* = 60). All patients had been diagnosed with IBD less than 5.7 years ago, with the exception of three patients who had been diagnosed with IBD 7.5–11.8 years earlier (Table 1).

### 3.2 Prevalence and severity of psychosocial and emotional problems

The mean score for total psychosocial problems was 10.8 (SD = 5.9), and for emotional problems 4.0 (SD = 2.5). Table 2 shows the prevalence and severity of psychosocial and emotional problems. Based on percentile scores, 45 of the 70 participants (64.3%) had elevated Total Difficulties scores and 30 (42.9%) had elevated Emotional Symptoms scores. Based on raw SDQ scores, adolescents with IBD reported significantly higher total difficulties than Dutch normative data, *t*(69) = 3.12, *p* = 0.003, *d* = 0.37, as well as significantly higher emotional symptoms, *t*(69) = 5.66, *p* < 0.001, *d* = 0.68.

**Table 2:** Severity and prevalence of psychosocial and emotional problems in adolescents with IBD.

	Mean (SD)	Norm mean (SD)	Elevated *n* (%) (≥80th percentile)
Total difficulties	10.8 (5.9)**	8.6 (5.0)	45 (64.3%)
Emotional symptoms	4.0 (2.5)***	2.3 (2.1)	30 (42.9%)

Abbreviations: IBD, inflammatory bowel disease; SD, standard deviation.

**p < 0.01

***p < 0.001.

### 3.3 Risk factors associated with psychosocial and emotional problems

To explore factors associated with psychosocial and emotional problems, Spearman correlations were first calculated (Table 3). Maladaptive ER strategies were associated with psychosocial (*r_s_* = 0.528, *p* < 0.001) and emotional problems (*r_s_* = 0.562, *p* < 0.001). Adaptive ER strategies were negatively associated with psychosocial problems (*r_s_* = −0.459, *p* < 0.001) and emotional problems (*r_s_* = −0.508, *p* < 0.001). Sex, age, SES, disease duration, disease activity, and CRM were not significantly correlated with either outcome (*p* > 0.05). Variables showing significant associations in the correlation analyses (Table 3) were then entered as candidate predictors in stepwise regression analyses (Table 4).

**Table 3:** Spearman correlations among demographic, disease‐related, and study variables.

Variables	1	2	3	4	5	6	7	8	9	10
1. Age	*‐*									
2. Sex	−0.20	‐								
3. SES	−0.03	−0.01	‐							
4. Disease duration	0.24	−0.24*	−0.03	‐						
5. Disease activity	−0.03	0.07	0.13	−0.45***	‐					
6. Maladaptive ER	0.44***	−0.37**	−0.07	0.19	0.03	‐				
7. Adaptive ER	−0.02	0.19	−0.14	−0.16	0.01	−0.42***	‐			
8. CRM	0.34**	−0.51**	−0.11	−0.38**	0.48**	0.43**	−0.26	‐		
9. Total difficulties	0.12	−0.21	0.13	0.13	0.07	0.53***	−0.46***	0.18	‐	
10. Emotional symptoms	0.17	−0.05	0.11	0.10	0.01	0.56***	−0.51***	0.14	0.75***	*‐*

*Note*: Correlations are Spearman's rho. Sex was coded as 0 (male) and 1 (female). Higher SES scores indicate higher SES. Disease activity is coded as an ordinal variable, with higher scores indicating higher disease activity. Higher scores on maladaptive and adaptive ER indicate more frequent use of these strategies. Higher CRM scores indicate greater cumulative risk exposure, calculated as the sum of dichotomized risk factors (0 = absence, 1 = presence). Psychosocial and emotional problems are based on raw Strengths and Difficulties Questionnaire scores, with higher scores indicating more problems.

Abbreviations: CRM, cumulative risk model; ER, emotion regulation; SES, socioeconomic status.

* *p* < 0.05

** *p* < 0.01

*** *p* < 0.001.

**Table 4:** Results of stepwise multiple regression analyses predicting total difficulties and emotional symptoms.

	*B*	SE	95% CI		*p*‐value
			LL	UL	
Total difficulties					
Constant	5.973	11.619	−17.285	29.231	0.609
Maladaptive ER strategies	1.508	0.272	0.962	2.053	<0.001
Emotional symptoms					
Constant	22.616	10.674	1.251	43.982	0.038
Maladaptive ER strategies	1.231	0.250	0.730	1.733	<0.001

*Note*: Only predictors retained in the final stepwise regression model are shown. Adaptive ER strategies were entered into the models but were not retained, as they did not contribute additional variance to the outcomes. *N* = 70. Abbreviations: CI, confidence interval; ER, emotion regulation; LL, lower limit; SE, standard error; UL, upper limit.

After the stepwise procedure, maladaptive ER strategies remained the only significant predictor of higher psychosocial and emotional problems. Adaptive ER strategies were not retained in the final models as they did not significantly contribute to the outcomes and are therefore not shown in Table 4. Maladaptive ER strategies remained a significant predictor of psychosocial and emotional problems in the final models (Table 4).

## 4 DISCUSSION

In this study, we investigated the prevalence of psychosocial and emotional problems, as well as the factors that may be associated with them, in adolescents with IBD. We found that psychosocial and emotional problems were significantly more prevalent in these individuals compared to the reference group. The comparison with Dutch normative data can be considered appropriate, as the reference data were based on self‐report SDQ scores from adolescents in the same age range (12–17 years). This supports the interpretability of the observed differences, with a substantially higher proportion of adolescents in the current sample showing elevated psychosocial (64.3%) and emotional problems (42.9%) than would be expected based on population norms. Part of this difference may be explained by differences in sample characteristics, as the current sample included a relatively higher proportion of girls and older adolescents compared with the Dutch reference sample.^29^ Previous research has shown that psychosocial and emotional problems tend to increase during adolescence and are more frequently reported by girls. Furthermore, recent studies have confirmed elevated rates of psychosocial problems in adolescents with IBD, supporting our findings.^39^, ^40^ Moreover, the elevated prevalence may reflect the additional psychosocial burden of living with a chronic disease, affecting daily life and well‐being. To further understand factors associated with these elevated rates, we examined the role of ER. Our findings indicate that adolescents with dysfunctional ER strategies (i.e., more maladaptive and fewer adaptive ER strategies) reported significantly more psychosocial and emotional problems.

This study adds to previous studies showing more psychological problems in adolescents with IBD^5^; however, the current finding should be interpreted with some caution, as we were unable to use an age‐ and sex‐matched control group, which limits direct comparison with peers. In addition, some SDQ items refer to somatic complaints, which may overlap with IBD symptoms. However, the SDQ also captures broader psychosocial domains, suggesting that higher scores likely reflect overall psychosocial functioning rather than physical symptoms alone. To our knowledge, our study is the first to show that dysfunctional ER is associated with psychosocial and emotional problems in adolescents with IBD. In particular, maladaptive ER strategies (e.g., giving up, withdrawal, aggressive behavior, self‐devaluation, and rumination) are strongly associated with psychosocial and emotional problems. The psychosocial functioning of adolescents is strongly linked to the use of ER strategies, both in the general population^41^ and in other chronic disease groups.^20^ Our study extends this finding to adolescents with IBD, highlighting the relevance of ER strategies and for psychosocial functioning in this population as well.

In the present study, additional demographic and clinical characteristics were not significantly associated with psychosocial and emotional problems. This finding differs from previous studies,^14^ this may be due to variables such as disease activity and disease duration showing limited variability, and it is possible that such associations would have been found in a larger sample. Given that demographic and clinical characteristics were not associated with psychosocial and emotional problems, it is understandable that a cumulative model including these risk factors was also not associated.

The “brain–gut” hypothesis, describing bidirectional interactions between IBD and psychological problems, may contribute to the higher prevalence of psychosocial and emotional difficulties. Psychological stress and maladaptive ER strategies can influence intestinal inflammation, while chronic inflammation and disease symptoms may affect psychological well‐being via immune and microbiota‐related pathways. However, the underlying mechanisms remain poorly understood.^42^ These interactions may partly explain why adolescents with IBD experience elevated psychosocial and emotional problems.

This study is limited by its cross‐sectional design, meaning that no conclusions can be drawn regarding the directionality of effects. In addition, the lack of an age‐ and sex‐matched control group represents a major limitation. It also remains unclear whether this group of adolescents differed from healthy peers prior to the onset of the disease. Although a clear association was found between the use of ER strategies and psychosocial and emotional problems, longitudinal research is required to establish the temporal nature of this relationship. Furthermore, a longitudinal design may provide greater insight into the mechanisms underlying the brain–gut hypothesis and their contribution to psychosocial and emotional problems. For future research, it would be valuable to request the SDQ data (completed as part of the standard screening procedure in youth healthcare) that was collected at age 10. Longitudinal comparison of pre‐ and post‐morbid information will enhance our understanding of the underlying mechanisms.

Psychosocial and emotional problems were measured using the SDQ. The SDQ is a brief instrument that has been well‐implemented and has proven to be highly useful. However, it could also be of interest to use a disease‐specific questionnaire, which may be more sensitive to the needs of these adolescents.^43^ Moreover, to obtain a more comprehensive overview, it is suggested that parent reports be included, with the objective of providing a complementary dimension to child reports. As research has shown, the inclusion of parent reports could potentially have resulted in higher proportions of psychosocial problems.^28^, ^29^

This study has important clinical implications. The SDQ and FEEL‐KJ may be useful tools for screening adolescents with IBD for elevated psychosocial and emotional problems, with high scores indicating the need for psychosocial assessment and, if needed, referral to mental health professionals. Routine screening also allows early identification of maladaptive ER strategies, facilitating timely intervention.^44^ Enhanced understanding of these strategies can guide early psychological interventions, such as psychoeducation and mindfulness. The psychosocial group program Op Koers, designed for children with chronic illness and their families, may be an effective intervention for adolescents with IBD.^45^ Because these strategies are modifiable, early intervention may support the development of adaptive coping strategies, reduce symptom severity, and prevent future psychosocial problems.

## 5 CONCLUSION

This study showed that adolescents with IBD have more psychosocial and emotional problems than their peers. These problems were strongly associated with dysfunctional, particularly maladaptive, ER strategies. We advise future researchers to use a longitudinal design to gain a fuller understanding of this association. Early identification and targeted interventions, such as psychoeducation or mindfulness, may help strengthen adaptive ER strategies and prevent future psychosocial difficulties in this vulnerable population.

## CONFLICT OF INTEREST STATEMENT

The authors declare no conflicts of interest.

## Supporting information

Supporting File 1.
