# Supplementary material for: Impact of emotion regulation on psychosocial and emotional problems in Dutch adolescents with inflammatory bowel disease
Source: JPGN Rep. 2026 Nov 3:10.1002/jpr3.70299. Online ahead of print. doi: 10.1002/jpr3.70299 (PMC13729237; doi:10.1002/jpr3.70299)
Supplement: Supplementary file 1 [file JPR3-9999-0-s001.docx]

| **Supplementary Table S1**. Overview of the Strengths and Difficulties Questionnaire (SDQ) | | |
| --- | --- | --- |
| Subscale (5 items each) | Description | Scoring (3-point Likert scale) |
| Emotional Symptoms | Items reflecting feelings of sadness, anxiety, or worry | 0 = Not true; 1 = Somewhat true; 2 = Certainly true |
| Conduct Problems | Items reflecting rule-breaking or aggressive behavior | 0 = Not true; 1 = Somewhat true; 2 = Certainly true |
| Hyperactivity/Inattention | Items reflecting restlessness, distractibility, or impulsivity | 0 = Not true; 1 = Somewhat true; 2 = Certainly true |
| Peer Problems | Items reflecting social difficulties with peers | 0 = Not true; 1 = Somewhat true; 2 = Certainly true |
| Prosocial Behavior | Items reflecting helpfulness, empathy, and cooperation | 0 = Not true; 1 = Somewhat true; 2 = Certainly true |

**Notes:**
*Total Difficulties* were calculated as the sum of the first four SDQ subscales (Emotional Symptoms, Conduct Problems, Hyperactivity/Inattention, Peer Problems), excluding Prosocial Behavior (range 0-40).
*Emotional Symptoms* were calculated as the sum of the Emotional Symptoms subscale (range 0-10).
Higher scores indicate more difficulties.
For more detailed description and validation of the SDQ, see Goodman (26) and recent Dutch norm studies (27, 28).
